# Initial motility and vitality predict the semen quality after long‐term cryostorage, even in patients with restricted ejaculate parameters

**DOI:** 10.1111/andr.70019

**Published:** 2025-03-06

**Authors:** Simone Bier, Daniela Hanke, Michael Zitzmann, Sabine Kliesch, Verena Nordhoff

**Affiliations:** ^1^ Department of Clinical and Surgical Andrology Centre of Reproductive Medicine and Andrology University Hospital of Münster Münster Germany

**Keywords:** long‐term cryostorage, male fertility protection, semen cryopreservation

## Abstract

**Background:**

Cryopreservation of human semen is the cornerstone for preserving male fertility before gonadotoxic therapy or in cases of high variability in semen parameters. This is particular crucial in cases of severe oligoasthenoteratozoospermia (OAT), where diminished sperm counts may compromise planned intracytoplasmic sperm injection (ICSI) procedures. Previous investigations in donor programs have shown long‐term storage effects, such as decreased motility in cryopreserved semen samples. However, these studies were based on patients exhibiting normozoospermic semen samples. To date, there has been no comprehensive evaluation of the effect of long‐term cryostorage on sperm samples from individuals with compromised semen parameters.

**Objectives:**

The aim of this study was to identify the effect of long‐term cryostorage on semen parameters such as motility and vitality. Additionally, we sought to identify variables, which could aid in predicting motility and vitality following the freeze‐thaw process.

**Patients and methods:**

Within our center, we have archived sperm samples from 6022 patients cryopreserved between 2001 and 2019. Among these, 293 patients donated their samples for subsequent research following depot termination. We examined semen concentration, motility, morphology, and vitality of spermatozoa thawed after varying storage durations, alongside baseline metrics documented at the time of cryopreservation. Samples were stratified into three cohorts based on storage duration: 2.5 to ≤5 years, > 5 to ≤14 years, and > 14 years.

**Results:**

Our analysis revealed no changes in motility (*p* = 0.44), vitality (*p* = 0.08), or morphology (*p* = 0.44) across the cohorts. Regression analysis demonstrated that initial motility and sperm concentration were significantly associated with post‐cryostorage motility, whereas storage duration was not (*p* = 0.72). Similarly, there was no association between storage duration and post‐thaw value 2 vitality (*p* = 0.64).

**Discussion:**

The initial semen analysis as well as the evaluation of a short‐term frozen sample immediately after cryopreservation, appeared to be the most important markers for predicting post‐thaw motility and vitality.

**Conclusion:**

Our results demonstrate the reliability of long‐term cryostorage of human spermatozoa for fertility preservation, even in individuals with constrained semen quality at the time of cryopreservation.

## 1 INTRODUCTION

Cryopreservation of human spermatozoa is the standard for preserving fertility in male cancer patients, those at risk of gonadotoxic therapy, patients with bladder neck obstruction, as well as men with severe oligozoospermia or ejaculatory disorders.^1^, ^2^, ^3^, ^4^, ^5^ Cryopreservation was first implemented in the 1960s for successful sperm storage and fertility preservation.^6^ A significant number of live births has been achieved using cryopreserved semen, facilitated by intracytoplasmic sperm injection (ICSI) or intrauterine insemination, depending on the quantity and motility of the stored ejaculates.^7^

To cryopreserve human spermatozoa, two techniques are employed: the traditional slow freezing method^8^, ^9^ and vitrification, a method adopted several years ago.^7^ Both methods affect spermatozoa, particularly the structural and functional integrity, due to extreme temperature changes from the body's 37°C to −196°C in liquid nitrogen. The freezing process, which poses risks such as dehydration and intracellular ice formation, and thawing process, where melting ice dilutes extracellular solutes causing water influx into the spermatozoa, are particularly critical phases.^10^ The success of cryopreservation depends partly on the initial quality of the semen and, as expected, on the techniques chosen for freezing and thawing.^11^, ^12^

After cryopreservation, adverse effects such as reduced motility and vitality, mitochondrial damage, and increased DNA fragmentation can be observed in 25–75% of spermatozoa.^13^ To forecast the behavior of a specific sample, an analysis of sperm motility and survival can be conducted 24 h after the initial cryopreservation step by thawing an additional frozen aliquot.^11^, ^12^ This examination aims to predict the quality of the thawed spermatozoa when the patient decides to use their stored sample for medically assisted reproduction (MAR).^13^, ^14^

Previous studies showed a storage‐dependent decline in motility in normozoospermic samples, which did not impact clinical pregnancy rates within this cohort.^15^ Encouragingly, other research, such as that by Kläver et al. (2012), indicates that sperm DNA methylation remains stable through cryopreservation. However, this study also confirmed a detrimental effect of extended cryostorage on motility.^16^

To date, the long‐term effects of cryostorage on frozen‐thawed spermatozoa from patients with oncological conditions or restricted sperm counts have not been thoroughly investigated. This study aimed to determine whether the duration of cryostorage influenced the motility and the vitality of the semen samples after storage and thawing, particularly in patients with limited semen parameters. Additionally, we wanted to identify variables which could be used to predict motility and vitality value due to both storage and the freeze‐thaw process.

## 2 MATERIALS AND METHODS

**FIGURE 1:**
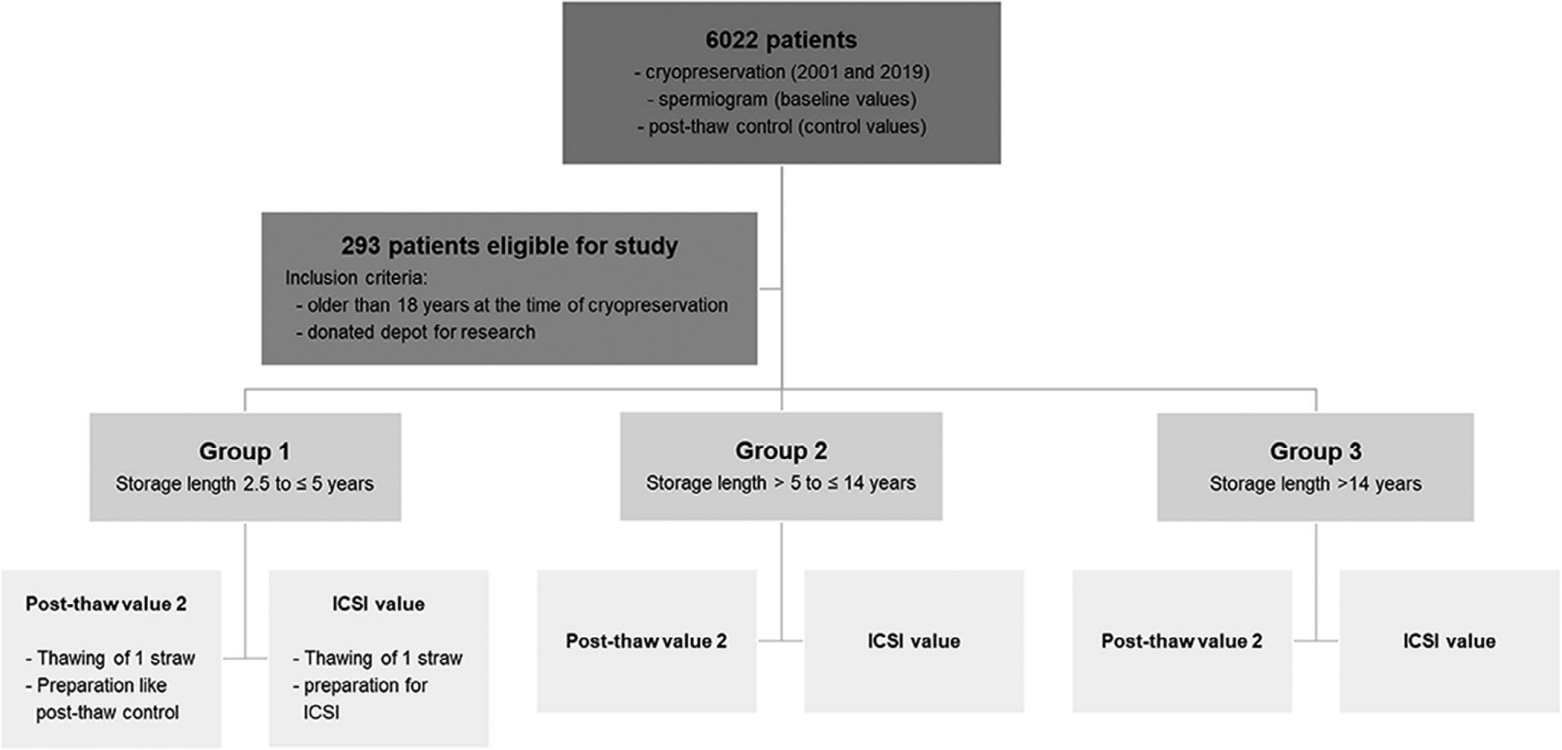
Flowchart represents patient recruitment for cryopreservation of semen, grouping of the patients according to storage duration and the method of thawing for this study. (ICSI: intracytoplasmic sperm injection).

The study received approval from the Institutional Review Board (trial no. 2021‐018‐f‐S), and informed consent was obtained from each patient. We analyzed semen samples from 293 patients who opted for cryopreservation, primarily for fertility preservation reasons. These patients donated their samples for research after either completing their family planning or experiencing a recovery of their semen parameters following potentially gonadotoxic therapy (Figure 1).

Patients with severe cryptozoospermia (fewer than 100,000 spermatozoa) were excluded due to the high risk of significant counting errors caused by the extremely low sperm counts, as were patients with azoospermia. We categorized our samples based on their storage duration: Group 1 comprised samples cryopreserved up to 5 years ago (cryostorage duration from 2.5 to 5 years, median: 4.0 years), Group 2 included samples cryopreserved between 5 and 14 years ago (cryostorage time ≥5 to 14 years, median: 9.8 years), and Group 3 consisted of samples cryopreserved for more than 14 years (cryostorage time > 14 years; median: 15.0 years) (Figure 1).

We collected general patient parameters, including smoking status, BMI, and testosterone values. Before cryopreservation the HIV and hepatitis status were evaluated in accordance with the requirements outlined in the WHO manual.^8^, ^9^

### Semen analysis before cryopreservation (baseline values)

Semen analysis was conducted in line with the World Health Organization (WHO) recommendations from the years 1999^17^ and 2010.^8^ This was necessary because the semen analysis for the long‐term storage group was performed before the publication of the WHO 2010 manual. Briefly, sperm concentration was measured in duplicates of 10 µL each using a hemocytometer. Motility assessment involved placing two 10 µL samples of native ejaculate under a 22 mm × 22 mm coverslip and 200 spermatozoa were counted per sample. Motile spermatozoa were categorized into: (1) progressive motile (PR), which includes fast progressive (a) and slow progressive (b), (2) non‐progressive (NPR as per WHO 2010 or “c” according to WHO 1999), and immotile spermatozoa (IM as per WHO 2010 or “d” according to WHO 1999). Vitality was assessed using eosin red staining, and sperm morphology was evaluated using Papanicolaou staining to establish baseline values.

### 2.1 Cryopreservation and evaluation of the freezing process

**FIGURE 2:**
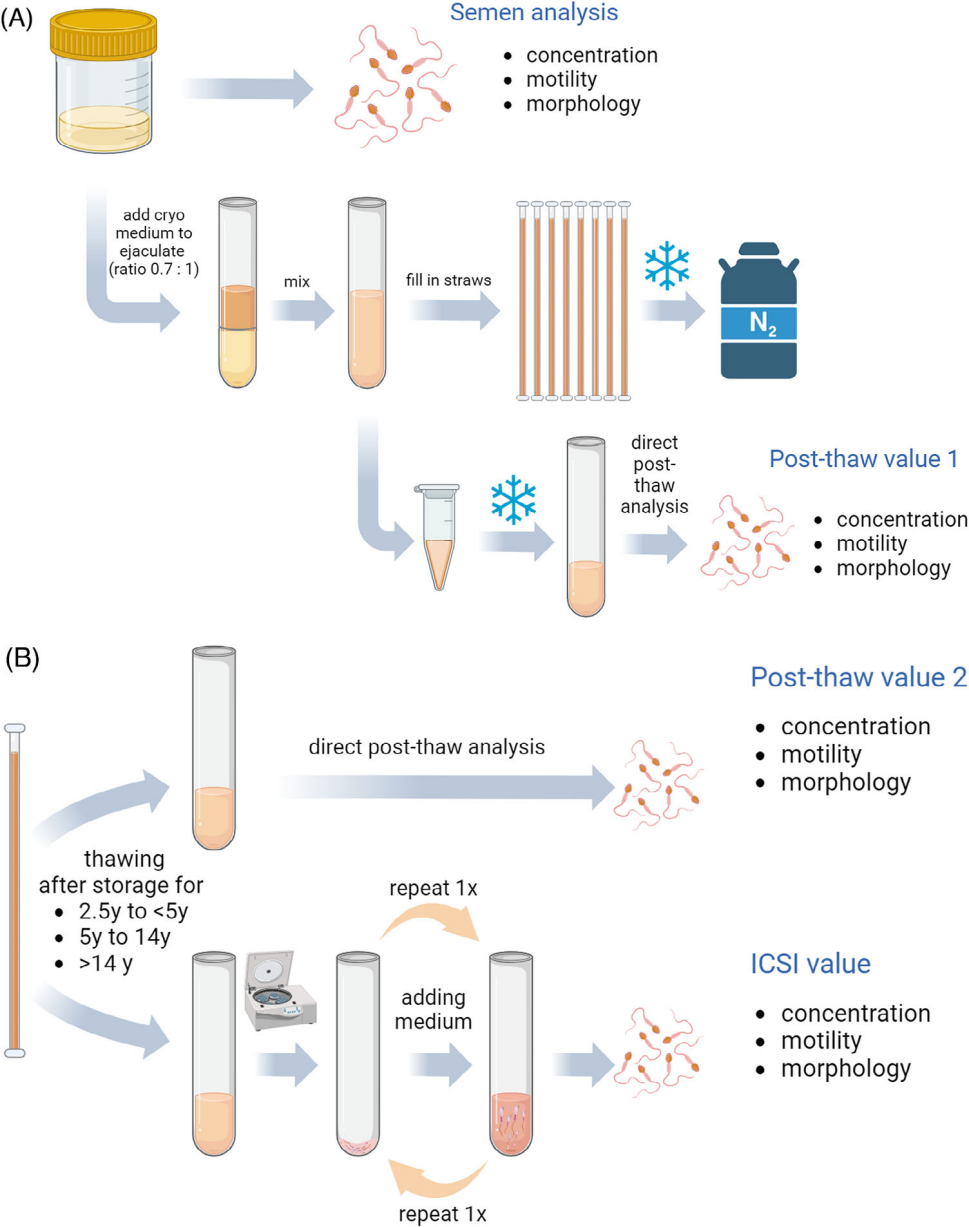
(A) Processing of the ejaculate for cryopreservation on the day of the initial visit, including the assessment of baseline parameters (concentration, motility, and morphology), the freezing procedure (middle row) and the thawing process for the “Post‐Thaw‐value 1”. (Figure created with BioRender). (B) Thawing process depending on the method of thawing: direct post thaw analysis for Post‐thaw value 2, or the washing step for preparation of ICSI value (ICSI: intracytoplasmic sperm injection, y: years). (Figure created with BioRender).

For cryopreservation, the ejaculate was diluted and gradually mixed with a prewarmed (37°C) cryoprotective solution (untill 2013: Steritec; SteriPharm Ltd, since 2013: CryoSperm™, Origio) in a 1:0.7 ratio (1 mL semen, 0.7 mL cryoprotectant). After gentle mixing, the solution was left at room temperature for 10 min for equilibration. This mixture was then aliquoted into single 0.25 mL straws (Minitüb), with an additional tube reserved for subsequent evaluation of the freezing process (Figure 2A). Each straw was sealed at both ends and cryopreserved using a computer‐operated automated freezer (Ice Cube 14S, Sy‐Lab) until it reached –170°C after 25 min (cryopreservation protocol in Figure S1). The straws were stored in designated cassettes (Minitüb) within a monitored storage tank equipped with an automated refill function (Ice Cube 14 S, Sy‐Lab).

To assess the freezing process and to analyze the future performance of the frozen sample on the day of MAR, the extra sample from the tube was thawed the day after freezing (referred to as post‐thaw value 1; Figure 2A). Without further preparation, the vitality and motility of the sample were determined using the same methods described above (e.g., eosin red staining for vitality). Motility was assessed in duplicates and documented in our patient management system, Androbase.^18^

### 2.2 Thawing of semen samples for evaluation of varying storage times

We employed two distinct methods to thaw our samples, as illustrated in Figure 2B. Initially, to generate a comparable value to the freezing evaluation conducted immediately after cryopreservation in previous years (referred to as the post‐thaw value 1), we thawed one of the donated straws after varying storage durations. This straw was analyzed for vitality and motility upon thawing, without any further handling, to determine the post‐thaw value 2.

However, the thawing process for samples intended for use in an ICSI cycle differed from the post‐thaw value processing. To simulate the ICSI preparation, the following procedure was adopted (referred to as the ICSI value, Figure 2B): a straw was thawed at room temperature for 15 min. The contents were then transferred to a round‐bottom 14 mL tube (Falcon) and centrifuged at 390 g for 10 min. The supernatant was gently discarded, and 2 mL of sperm preparation medium (Cat. No. 10700060, Origio), pre‐equilibrated at 37°C and 5% CO_2_ in air, was added and gently mixed. The mixture was then centrifuged again at 390 g for 10 min. Subsequently, the supernatant was decanted, and 200 µL of fresh, equilibrated sperm preparation medium was added. Motility was assessed by taking 10 µL from the sperm preparation (as described earlier) and counting 100 spermatozoa in duplicates.

### 2.3 Statistical analysis

For the statistical analysis, we utilized SPSS software (version 27.0). To compare patient and semen characteristics in relation to the length of cryostorage, an ANOVA test was conducted. Additionally, a regression analysis was performed to evaluate potential influencing factors on the outcomes. Given the high variability of the ejaculate samples, we used the Wilcoxon signed‐rank test for repeated measurements within a single sample to evaluate the changes of the semen quality due to the storage duration.

## 3 RESULTS

Between 2001 and 2019, a total of 6022 patients preserved their semen samples at our center to maintain their fertility. Of these, 293 patients (> 18 years) terminated their storage and donated their samples for research; these were included in our study (Figure 1). The primary reasons for cryopreservation included malignancies (*n* = 217, 74%), testicular cancer (*n* = 98), leukemia (*n* = 5), sarcoma (*n* = 5), lymphoma (*n* = 27), gastrointestinal cancer (*n* = 6) and other tumors. Additionally, some patients preserved their samples for MAR (*n* = 51, 17%), hormonal disorders (*n* = 5, 2%), and other causes such as multiple sclerosis, Crohn's disease, other autoimmune diseases, and presurgical procedures like varicocele ligation (*n* = 20, 7%; Table 1).

**TABLE 1:** Outline of the various reasons for semen cryopreservation (MAR, malignancies, hormonal disorders and a range of other indications; for details see results section); groups are classified according to the length of storage (Group 1: 2.5–5 years; Group 2: 6–14 years; Group 3: > 14 years).

	MAR *n* [%]	Malignancies *n* [%]	Hormonal disorders*n* [%]	Others *n* [%]	Total
Group 1 Storage 2.5–5 year	12 [24]	30 [14]	1 [20]	6 [30]	49
Group 2 Storage 6–14 year	38 [74]	158 [73]	4 [80]	13 [65]	213
Group 3 Storage > 14 year	1 [2]	29 [13]	0 [0]	1 [5]	31
Total	51	217	5	20	293

Abbreviations: MAR: medically assisted reproduction; n: numbers.

### 3.1 Patient and Semen characteristics

Smoking status was evaluated in 238 of 293 patients. At the time of cryopreservation, 37% (*n* = 13) of the patients in Group 1, 32% of the patients in Group 2 (*n* = 55) and 36% of the patients in Group 3 (*n* = 11) smoked regularly. BMI data were available in 218 of 293 patients: the mean BMI was the same across all groups, with 26.2 ± 5.6 kg/m^2^ in Group 1, 26.2 ± 5.0 kg/m^2^ in Group 2 and 26.2 ± 4.8 kg/m^2^ in Group 3. Testosterone values were evaluated in 269 of the 293 patients at time of cryopreservation. There was a significant difference in the testosterone value between the groups (*p* = 0.004). In Group 1 the mean testosterone value was 18.5 ± 7.4 nmol/L, in Group 2 it was 15.7 ± 7.5 nmol/L, and in Group 3 it was 12.8 ± 4.8 nmol/L. However, the mean testosterone value in all groups was within the normal range.

At the time of cryopreservation, the median age of our 293 patients was 32.2 years, ranging from 18 to 62 years. The median sperm concentration at the time of cryopreservation was 15.4 million/mL, with a range from 0.1 to 418 million/mL. The total sperm count per ejaculate was 45.7 million, ranging from 0.2 to 2,173 million. For progressive motility (PR, categories a + b), the median was 47.9%, with a range from 0 to 71.0%.

There was a significant decline in progressive motility from the baseline (44.3 ± 12.7% PR) to the post‐thaw value 1 (25.0 ± 14.3% PR) following the freeze‐thaw process (*p *< 0.001). Additionally, the baseline vitality of spermatozoa was assessed in 212 patients at the time of cryopreservation, due to either a small volume of seminal fluid or very low sperm concentration. The mean baseline vitality was 63.5% ± 11.9%, which also significantly decreased compared to the post‐thaw value 1 vitality (45.0 ± 14.2%) after the freeze‐thaw process (*p* < 0.001).

Morphology was assessed in 240 samples. However, since strict criteria regarding morphology according to WHO recommendations were implemented starting in 2010, morphology values changed, making a comprehensive evaluation of all morphology values infeasible. To address this issue, we restricted our analysis to values collected before 2010. This approach resulted in 197 evaluated samples, of which 44% (47/197) displayed normal morphology at the time of cryopreservation.

### 3.2 Baseline characteristics according to the storage length

The baseline semen characteristics of patients on the day of cryopreservation were grouped according to storage duration and summarized in Table 2. No significant differences were observed in age or sperm parameters such as concentration, total sperm count, motility, and vitality before freezing among the three groups. Additionally, the post‐thaw value 1 showed consistent results across most parameters at the time of thawing; however, there was one exception: post‐thaw value 1 motility differed significantly between the groups, with the lowest values observed in Group 1 (Table 2).

**TABLE 2:** Detailed comparison of baseline semen characteristics (assessed at the time of cryopreservation) and post‐thaw control value 1 parameters (evaluated after the initial thawing process), grouped according to the duration of storage (Group 1: 2.5–5 years; Group 2: 6–14 years; Group 3: > 14 years). Key parameters such as sperm concentration, motility, vitality, and morphology were analyzed to determine any potential differences or trends related to storage length (data are presented as mean ± SD and median and range in brackets).

	Group 1 (n = 49) Storage time 2.5–5 years	Group 2 (n = 213) Storage time 6–14 years	Group 3 (n = 31) Storage time > 14 years	
	Mean ± SD (Median; Range)	Mean ± SD (Median; Range)	Mean ± SD (Median; Range)	*p*‐value
Age (years)	33.9 ± 7.1 (34.4; 19–47)	32.2 ± 7.9 (31.8; 18–62)	31.4 ± 5.5 (32.0; 21–46)	0.28
Sperm analysis on day of cryopreservation				
Concentration [Million/mL]	24.9 ± 34.7 (31.8; 0.1–156.5)	30.4 ± 38.5 (16.6; 0.1–188.0)	41.0 ± 87.4 (15.1; 0.8–418.0)	0.33
Sperm count [Million/ejaculate]	80.0 ± 127.2 (32.2; 0.2–735.6)	123.8 ± 193.4 (47.0; 0.2–1122.0)	189.5 ± 458.5 (43.0; 3.8–2173.0)	0.13
Vitality (baseline) [%]	66.3 ± 15.8 (68.0; 14–85) N = 31	64.8 ± 10.5 (66.5; 6–84) N = 157	59.3 ± 9.0 (61.0; 39–71) N = 24	0.051
Motility (baseline) [Progressive motility; a+b]	41.5 ± 14.2 (45.7; 5.0–62.0)	45.2 ± 12.7 (48.2; 0.0–71.0)	43.7 ± 10.7 (47.0; 8.0–56.5)	0.18
Post thaw control on the day after cryopreservation				
Vitality (post thaw value 1) [%]	46.0 ± 16.4 (46; 8–70)	43.8 ± 14.1 (47; 0–96)	39.0 ± 15.0 (34.5; 0–70)	0.17
Motility (post thaw value 1) [progressive motility, a+b]	20.5 ± 14.9 (20.0; 0–49.0)	25.9 ± 13.5 (27.1; 0–97.0)	23.7 ± 11.4 (22.8; 0–44.0)	**0.04**

### 3.3 Influence of storage length on motility and vitality

Regardless of the thawing procedure used for the samples—whether it was post‐thaw value 2 (one straw) or the ICSI value (one straw), there was no association of storage length and motility or vitality, as indicated in Table 3.

**TABLE 3:** Variations in motility, sperm vitality and progressive motility, grouped according to storage duration (Group 1: 2.5–5 years; Group 2: 6–14 years; Group 3: > 14 years) and the specific thawing method used—either preparation for ICSI or of post‐thaw value 2 preparation.

	Group 1 (*n* = 49) Storage time 2.5–5 years	Group 2 (*n* = 213) Storage time 6–14 years	Group 3 (*n* = 31) Storage time > 14 years	
	Mean ± SD (Median; Range)	Mean ± SD (Median; Range)	Mean ± SD (Median; Range)	*p*‐value
**ICSI value**				
Motility prepared for ICSI [Progressive motility, a+b]	14.7 ± 15.6 (12.0; 0–48.0)	13.6 ± 13.9 (8.7; 0–53.0)	17.3 ± 15.4 (15.0; 0–46.0)	0.44
**Post thaw value 2, after long‐term storage**				
Vitality (post thaw value 2) [%]	42.6 ± 18.5 (42; 3–70)	35.4 ± 15.7 (37.5; 18–91)	37.9 ± 19.2 (34.5; 0–70)	0.08
Motility (post thaw value 2) [Progressive motility, a+b]	17.8 ± 17.3 (13.7; 0–48.0)	16.1 ± 15.1 (11.3; 0–52.0)	20.0 ± 17.4 (16.0; 0–48.0)	0.44

*Note*: Data are presented as mean ± SD and median with range in brackets.

To compare the changes of the motility due to storage duration, we used the Wilcoxon Signed‐Rank Test to evaluate the progressive motility of post thaw value 1 against post thaw value 2. No significant changes in motility were observed across all groups (Group 1: *p* = 0.16, Group 2: *p* = 0.22, Group 3, *p* = 0.41).

### 3.4 Influencing factors on post‐thaw value 2 motility and vitality

To assess the potential influence of various factors on thawing outcomes, we conducted a regression analysis. This analysis compared the final motility values after long‐term cryostorage with baseline parameters such as sperm count, vitality, a+b motility, and storage length. We identified significant associations between baseline sperm count (*p* = 0.017) and baseline motility (*p* = 0.045). However, there is no association between storage length and post‐thaw value 2 motility (*p* = 0.85) or baseline vitality (*p* = 0.077). (Table 4).

**TABLE 4:** Regression analysis with the dependent variable being the post‐thaw value 2. This value was compared to sperm count, baseline vitality, baseline motility, and storage duration.

Variable	Standard error	Beta	*t*‐ratio	*p*‐value
Sperm count (mio)	.005	.273	2.461	0.094
Baseline vitality	.215	.291	1.806	**0.017**
Baseline motility (a+b)	.251	.301	2.055	0.077
storage duration	2.138	.023	0.183	**0.045**

*Note*: Parameters are presented in descending order according to p‐value; a+b: Progressive motility.

When comparing the motility values from the ICSI preparation to the baseline values, an association was observed between post‐thaw value 1 motility and ICSI motility (*p* = 0.001). However, no association was found between ICSI motility and storage length, sperm count, baseline motility or baseline vitality (Table 5)

**TABLE 5:** Regression analysis with the dependent variable being the motility prepared for ICSI. This value was compared to storage duration, sperm count, baseline motility, baseline vitality and post‐thaw values 1.

Variable	Std. error	Beta	*t*‐ratio	*p*‐value
Storage duration	2.388	0.008	0.058	0.954
Sperm count (mio)	0.005	0.015	0.134	0.894
Baseline motility (a+b)	0.245	0.065	0.470	0.641
Baseline vitality	0.221	0.132	0.826	0.413
Motility (post‐thaw value 1)	0.232	0.599	3.371	**0.001**

*Note*: Parameters are presented in descending order according to *p*‐value; a+b: Progressive motility.

In our regression analysis concerning post‐thaw value 2 vitality, there was no association with the storage length (*p *= 0.12) and sperm concentration (*p* = 0.057). However, several significant factors affecting post‐thaw value 2 vitality), baseline vitality (*p* = 0.001), post‐thaw control vitality (*p* = 0.005), and morphology (*p* = 0.006) were identified (Table 6).

**TABLE 6:** Regression analysis with the dependent variable: Post‐thaw value 2 vitality. This value was compared to storage duration, sperm concentration, morphology, post‐thaw value 1 vitality, and baseline vitality.

Variable	Std. error	Beta	t‐ratio	p‐value
Storage duration	2.162	−0.106	−1.526	0.129
Sperm concentration (mio/mL)	0.024	0.129	1.916	0.057
Morphology	0.262	0.207	2.783	**0.006**
Vitality (post thaw value 1)	0.085	0.203	2.814	**0.005**
0.00Baseline vitality	0.103	0.250	3.521	**0.001**

*Note*: Parameters are presented in descending order according to *p*‐value.

### 3.5 Results of patients with normal sperm count ≥ 39 million sperm count

To assess the effect of cryostorage on patients with a normal sperm count (≥39.0 million spermatozoa per ejaculate) at the time of cryopreservation, these individuals were categorized into the three before mentioned storage groups (Groups 1 to 3). Our analysis revealed no differences between groups in terms of age at cryopreservation (*p* = 0.70), baseline concentration (*p* = 0.36), baseline motility (*p* = 0.82), ICSI motility value (*p* = 0.50), or post‐thaw 2 motility (*p* = 0.22). However, post‐thaw value 1 motility showed a difference, though it was not statistically significant (*p* = 0.08; Table 4). These results indicate consistency in sperm quality post‐cryostorage across different storage durations, with slight variation in post‐thaw value 1 motility that may warrant further investigation (Table 7).

**TABLE 7:** Subgroup comparison among patients with a normal sperm count, ≥ 39 million spermatozoa (*n *= 153). The comparison included baseline semen characteristics, post‐thaw control values 1 and 2, as well as ICSI values, with the samples grouped according to the duration of storage (Group 1: 2.5–5 years; Group 2: 6–14 years; Group 3: > 14 years).

	Group 1 Storage 2.5–5 years	Group 2 Storage 6–14 years	Group 3 Storage > 14 years	
	Mean ± SD (Median; Range)	Mean ± SD (Median; Range)	Mean ± SD (Median; Range)	*p*‐value
**Variable**				
N	22	115	16	
Age	32.5 ± 7.4 (34.3; 19–46)	31.4 ± 8.0 (30.0; 19–62.0)	30.2 ± 5.2 (32.0; 22–36)	0.70
Concentration [million/mL]	50.0 ± 38.8 (42.3; 5.7–156,5)	51.0 ± 41.6 (37.6; 4.2–188.0)	72.2 ± 114.4 (31.5; 14.4–418.0)	0.36
Sperm count [Million]	166.0 ± 150.1 (123.5; 39.8–1122.0)	215.4 ± 222.7 (123.2; 39.8–1122.0)	347.3 ± 603.5 (139.4; 43.0–2173.0)	0.15
Motility (baseline) [Progressive motility, a+b]	49.1 ± 12.4 (54.0; 5.0–62.0)	50.0 ± 8.6 (50.7; 12.0–71.0)	48.6 ± 7.7 (51.3; 32.0–56.0)	0.82
Motility (post‐thaw value 1) [Progressive motility, a+b]	28.7 ± 11.7 (29.0; 2.0–47.0)	32.5 ± 11.5 (34.0; 0–51.0)	26.0 ± 12.6 (22.0; 1.0–44.0)	0.08
Motility prepared for ICSI [Progressive motility, a+b]	24.2 ± 15.3 (23.5; 0–48.0)	20.2 ± 14.1 (21.2; 0–53.0)	20.8 ± 16.8 (24.0; 0–46.0)	0.50
Motility (post‐thaw value 2) [Progressive motility, a+b]	29.1 ± 15.4 (31.0; 1.0–48)	23.0 ± 14.6 (24.2; (0–52.0)	24.8 ± 18.9 (29.0; 0–48.0)	0.22

*Note*: Data are presented as mean ± SD and median with range in brackets.

Regression analysis revealed that storage duration had no effect on ICSI motility (*p* = 0.19). However, a correlation was observed between initial total sperm count and ICSI motility (*p* = 0.05), indicating that higher initial sperm counts at the time of cryopreservation are associated with better motility outcomes in ICSI procedures.

### 3.6 Results of patients with oligozoospermia (total sperm count < 39 million per ejaculate)

A subgroup analysis was conducted on patients with oligozoospermia (total sperm count less than 39 million spermatozoa per ejaculate) who were further categorized on storage duration (Table 5). Interestingly, no significant differences were observed among the groups in terms of age (*p* = 0.36), sperm concentration (*p *= 0.56), total sperm count (*p* = 0.97), baseline motility, or post‐thaw value 1 motility (*p* = 0.38 and *p* = 0.19, respectively). Additionally, post‐thaw value 2 motility remained uniform across the three storage groups (*p* = 0.23). However, when samples were prepared for a virtual ICSI (ICSI value), a difference in motility was observed, with Group 3 demonstrating the most favorable outcome (*p* = 0.05; Table 8).

**TABLE 8:** Subgroup comparison among patients with oligozoospermia (sperm count < 39 million) (*n* = 140). The comparison included baseline semen characteristics, post‐thaw control values 1 and 2, as well as ICSI values, with the samples grouped according to the duration of storage (Group 1: 2.5–5 years; Group 2: 6–14 years; Group 3: > 14 years).

	Group 1 Storage 2.5–5 years	Group 2 Storage 6–14 years	Group 3 Storage > 14 years	
	Mean ± SD (Median; Range)	Mean ± SD (Median; Range)	Mean ± SD (Median; Range)	*p*‐value
**Variable**				
N	27	98	15	
Age	35.2 ± 6.6 (34.7; 21–47)	33.3 ± 7.8 (33.6; 18–57)	31.8 ± 6.1 (33.4; 18–57)	0.36
Concentration [million/mL]	4.4 ± 6.1 (0.8; 0.1–24.3)	5.4 ± 6.8 (3.1; 0.1–39.2)	6.7 ± 4.4 (3.2; 0.1–39.2)	0.56
Sperm count [million]	9.2 ± 11.5 (3.3; 0.2–38.4)	12.5 ± 11.1 (9.4; 0.2–38.9)	17.4 ± 11.0 (8.9; 0.2–39.0)	0.97
Motility (baseline) [Progressive motility, a+b]	35.3 ± 12.6 (34.8; 6.0–56.0)	39.4 ± 14.4 (42.2; 0–70.0)	37.5 ± 11.2 (40.3; 0–70.0)	0.38
Motility (post thaw value 1) [Progressive Motility, a+b]	13.8 ± 14.1 (8.0; 0–49.0)	19.1 ± 13.9 (18.4; 0–97.0)	19.6 ± 10.4 (17.8; 0–97.0)	0.19
Motility prepared for ICSI [Progressive motility, a+b]	7.1 ± 11.2 (0.81; 0–39.0)	5.6 ± 8.6 (1.3; 0–36.0)	12.7 ± 13.2 (1.6; 0–39.0)	**0.05**
Motility (post thaw value 2) [Progressive motility, a+b]	8.6 ± 12.9 (0.9; 0–42.0)	7.8 ± 11.0 (3.1; 0–42.0)	13.8 ± 14.6 (2.9; 0–45.0)	0.23

*Note*: Data are presented as mean ± SD and median with range in brackets.

The regression analysis within this subset found no evidence of storage duration influencing ICSI motility (*p* = 0.38), and baseline motility had no effect on ICSI motility (*p* = 0.43). Surprisingly, only baseline concentration had a measurable effect on ICSI motility values (*p* = 0.008). Further analysis revealed that solely concentration and baseline motility correlated significantly with post‐thaw 2 values (*p* = 0.02 and *p* = 0.01, respectively), whereas storage duration had no detectable effect (*p* = 0.99).

## 4 DISCUSSION

The results of our study highlight the importance of the initial semen quality in determining the vitality and motility of spermatozoa after the thawing process. Our findings suggest that, for patients in fertility‐compromising situations, the quality of the initial semen sample is a pivotal factor influencing the potential success of subsequent fertility treatments.

In this study, we examined storage durations exceeding 15 years, a valid consideration, particularly for young cancer patients who may need to cryopreserve semen for extended periods. Advances in malignancy therapy have significantly improved survival rates and quality of life, enabling many patients to pursue family‐building goals even years after their primary treatment. Consequently, understanding the effects of prolonged cryostorage on semen quality is of paramount importance.^19^

Our findings revealed no significant effect of storage duration on sperm vitality or motility, regardless of the thawing technique employed. However, we identified a correlation between baseline parameters (concentration, motility, and vitality) and post‐thaw characteristics (motility and vitality), which ultimately influenced final outcomes. This highlights the importance of baseline assessments in predicting post‐thaw performance. Furthermore, the results emphasize the value of post‐thaw evaluations in estimating motility outcomes immediately after cryopreservation. These assessments are instrumental in guiding patients or couples toward the most suitable reproductive interventions, whether conventional intrauterine insemination, in vitro fertilization, or ICSI—thereby ensuring personalized and informed fertility treatment plans.

Consistent with existing literature^15^, ^16^, ^20^, ^21^ we observed a significant decline in both motility and vitality following the freeze‐thaw process of cryopreserved samples. Notably, baseline motility exhibited no variance among the three groups. Interestingly, however, post‐thaw control motility in Group 2, representing storage durations of 5–14 years, surpassed that of Groups 1 and 3, which corresponded to storage durations less than 5 years and more than 14 years, respectively. It is important to highlight that there were no personnel changes or alterations in protocols during this timeframe. The only procedural difference was the adoption of a new cryopreservation medium in 2013. Despite this change, both Groups 1 and 2 underwent cryopreservation using the same medium, yet Group 1 displayed no significant differences in sperm parameters.

Huang et al. (2019) demonstrated that storing donor spermatozoa for more than 5 years negatively affected motility in a select group of normozoospermic samples.^15^ In contrast, our study did not detect any decline in motility values in post‐thaw value 2 parameters or in the preparations mimicking the conditions on the day of ICSI. One critical difference lies in the maintenance protocols for storage containers. Unlike the yearly cleaning procedures described by Huang et al. (2019), which involved exposing cryopreserved spermatozoa to room temperature, our center follows a more conservative protocol. Cryopreserved spermatozoa are only retrieved from the storage tank when they are required for use, ensuring that samples remain undisturbed and protected from environmental temperature fluctuations.^15^ This approach provides reassurance to young cancer patients that their stored spermatozoa are safeguarded against declines in motility, even during prolonged storage durations.

Regarding vitality, we detected no variance in baseline vitality among the three groups, nor did we observe any differences in post‐thaw value 1 vitality. Similarly, the final post‐thaw value 2 vitality showed no significant deviations in relation to storage duration. It is well‐established that the freeze‐thaw process leads to a significant decline in vitality, primarily due to the formation of ice crystals—a phenomenon not entirely mitigated by the use of cryoprotectants.^22^ Nevertheless, a case study published in 2013 presented compelling evidence to the contrary. The study reported the successful birth of twins following ICSI using semen cryopreserved for 40 years.^23^ Such remarkable outcomes highlight the resilience of cryopreserved spermatozoa and challenge long‐standing assumptions regarding the limitations of cryopreservation over extended storage durations.

Our regression analysis identified baseline vitality, along with baseline concentration and post‐thaw value 1 vitality, as reliable predictors of the expected final vitality. Similarly, baseline concentration, baseline motility, and post‐thaw value 1 vitality demonstrated strong predictive capabilities for outcomes on the day of ICSI, irrespective of storage duration. Notably, our findings diverge from studies in which baseline semen parameters fell within the normal range. Such studies often involved animal semen used for research purposes or human sperm samples from donor banks, which are typically normozoospermic.^15^, ^24^, ^25^ However, it is widely recognized that successful pregnancies can be achieved using long‐term cryopreserved semen in assisted reproductive technology (ART), aligning with our results.^23^, ^26^

After stratifying our cohort based on sperm count, dividing patients into those with a normal sperm count (≥39.0 million/ejaculate) and those with oligozoospermia (< 39.0 million/ejaculate), and further categorizing them into the three storage length groups, we repeated the statistical analysis to evaluate potential outcomes for the day of ICSI. Interestingly, in both the normozoospermic and oligozoospermic groups, our regression analysis did not identify any significant effect of storage length on motility. However, within the oligozoospermic cohort, a notable observation emerged: higher ICSI motility was recorded in Group 3 (storage length > 14 years) compared to the other two groups. Despite this, our regression analysis emphasized that baseline concentration and baseline motility significantly influenced ICSI motility, irrespective of storage duration. These findings suggest that, while storage length may not directly impact motility outcomes, baseline semen parameters play a pivotal role in determining the ICSI success, particularly in patients with oligozoospermia.

Our study examined the impact of long‐term storage on cryopreserved sperm samples from patients who underwent fertility preservation, many of whom did not exhibit normal baseline semen parameters. Our findings are exceptionally encouraging, as storage duration demonstrated no discernible influence on sperm parameters such as vitality and motility. Remarkably, even among patients with suboptimal ejaculate quality, we found no evidence that storage duration affected sperm quality. However, it is worth noting that favorable baseline characteristics were associated with better post‐storage quality, irrespective of storage duration. Consequently, both prefreezing ejaculate evaluation and post‐thaw control assessment of individual straws are indispensable for providing patients with accurate expectations regarding their future family planning. Our results not only provide reassurance to patients undergoing fertility preservation but also underscore the importance of comprehensive semen evaluation throughout the cryopreservation process. This ensures informed decision‐making and realistic counseling for patients embarking on their reproductive journey. However, it is important to acknowledge that only a small number of patients had storage duration exceeding 14 years. Furthermore, only patients with fulfilled desire for children or normal ejaculate parameters following their therapy provided their sample for research. Therefore, further studies–particularly involving larger cohorts with a wider range of tumor entities—are warranted. Additionally, future research could explore e.g. the analysis of HPV in the semen, which may provide further insights.^27^

In summary, this study is the first investigation into the impact of storage duration on sperm samples from patients with varying baseline semen parameters, including those with suboptimal ejaculate quality. Regardless of storage length, good baseline semen characteristics are strongly associated with better post‐storage quality. Moreover, long‐term storage of cryopreserved sperm samples for fertility preservation does not significantly affect parameters such as vitality and motility. These findings provide reassurance to patients undergoing fertility preservation and highlight the critical role of thorough semen evaluation throughout the cryopreservation process.

## AUTHOR CONTRIBUTIONS

Simone Bier designed the study, did the experiments and wrote the manuscript. Daniela Hanke did the experiments. Michael Zitzmann did the statistical analysis and wrote the manuscript. Sabine Kliesch designed the study and wrote the manuscript. Verena Nordhoff designed the study and wrote the manuscript. All authors approved the manuscript.

## CONFLICT OF INTEREST STATEMENT

The authors declare no conflicts of interest.

## Supporting information



Supporting Information

## Data Availability

The data that support the findings of this study are available from the corresponding author upon reasonable request.
