# Supplementary material for: Initial motility and vitality predict the semen quality after long‐term cryostorage, even in patients with restricted ejaculate parameters
Source: Andrology. 2025 Mar 6;13(8):2131–41. doi: 10.1111/andr.70019 (PMC12569725; doi:10.1111/andr.70019)
Supplement: Supplementary file 1 — Supporting Information [file ANDR-13-2131-s001.docx]

Supplementary Figure S1

Cryopreservation program for ejaculate using the Ice Cube 14S, Sy-Lab (Neupurkersdorf, Austria) (sec: seconds, min: minutes).

1. keep samples at +21°C for 5 min

2. rate of -4°C in 12 sec until +17°C

3. rate of -1°C in 4 sec until +16°C

4. rate of -4°C in 22 sec until +12°C

5. rate of -22°C in 2min 45sec until -10°C

6. rate of -60°C in 2 min 50 sec until -70°C

7. rate of -30°C in 2 min 20 sec until -100°C

8. rate of -10°C in 1 min 25 sec until -110°C

9. rate of -2,9°C in 23 sec until -110°C

10. rate of -56°C in 2min 15 sec until -170°C

11. plunge into liquid nitrogen and transfer to storage tank
